# Measuring the quality of transitional care based on elderly patients’ experiences with the partners at care transitions measure: a cross-sectional survey

**DOI:** 10.1186/s12912-024-01847-7

**Published:** 2024-03-14

**Authors:** La-Mei Liu, Meng-Yao Zhuansun, Tong-Yao Xu, Yu-Meng Qian, Hui-Qin Zhang, Qi-Han Zhang, Yi-Zhen Zhang

**Affiliations:** 1 School of Nursing and Health, Zhengzhou University, 100 Science Avenue, High-tech District, 450000 Zhengzhou City, Henan Province China; 2 Tongji Shanxi Hospital, Shanxi Bethune Hospital, Shanxi Academy of Medical Sciences, Third Hospital of Shanxi Medical University, 030032 Taiyuan City, Shanxi province China

**Keywords:** Older people, Chronic diseases, Transitional care, Quality of care, Influencing factors

## Abstract

**Background:**

The quality of transitional care is closely related to the health outcomes of patients, and understanding the status of transitional care for patients is crucial to improving the health outcomes of patients. Therefore, this study aims to investigate the quality of transitional care in elderly patients with chronic diseases and analyze its influencing factors, to provide a basis for improving transitional care services.

**Methods:**

This is a cross-sectional study. We used the Chinese version of the Partners at Care Transitions Measure (PACT-M) to survey patients with chronic diseases aged 60 years and older who were about to be discharged from five tertiary hospitals in Henan and Shanxi provinces. We used the mean ± standard deviation to describe the quality of transitional care, t-test or one-way ANOVA, and regression analysis to explore the factors affecting the quality of transitional care for patients.

**Results:**

182 elderly patients with chronic diseases aged ≥ 60 years completed the PACT-M survey. The scores of PACT-M_1_ and PACT-M_2_ were (30.69 ± 7.87) and (25.59 ± 7.14) points, respectively. The results of the t-test or one-way ANOVA showed that the patient’s marital status, ethnicity, religion, educational level, preretirement occupation, residence, household income per month, and living situation had an impact on the quality of transitional care for elderly patients with chronic diseases (*P* < 0.05). The results of regression analyses showed that patients’ preretirement occupation, social support, and health status were the main influences on the quality of transitional care for elderly patients with chronic diseases (*P* < 0.05), and they explained 63.1% of the total variance.

**Conclusions:**

The quality of transitional care for older patients with chronic illnesses during the transition from hospital to home needs further improvement. Factors affecting the quality of transitional care included patients’ pre-retirement occupation, social support, and health status. We can improve the hospital-community-family tertiary linkage service to provide coordinated and continuous transitional care for patients based on their occupation, health status, and social support to enhance the quality of transitional care and the patient’s health.

**Supplementary Information:**

The online version contains supplementary material available at 10.1186/s12912-024-01847-7.

## Background

China has the world’s largest elderly population, with a total of 280.04 million individuals aged 60 years and above, accounting for 19.8% of the overall population, and 209.78 million individuals aged 65 years and above, accounting for 14.9% of the total population by the end of 2022 [1]. The aging of the population and changes in people’s lifestyles have led to the emergence of chronic diseases as a significant public health concern that poses threats to the lives and well-being of individuals in China [2]. The prevalence of chronic diseases among older individuals exceeds 180 million, with a staggering 75% suffering from one or more such conditions. Moreover, the co-occurrence of multiple chronic diseases is highly prevalent in the elderly population [3]. Chronic diseases are long-term, recurring, and prolonged, requiring frequent travel to and from the hospital, community, and home settings to access different levels of care [4]. Transitional care encompasses a broad range of services and environments designed to promote the safe and timely passage of patients between levels of health care and across care settings [5]. The provision of high-quality transitional care is particularly crucial for elderly patients with chronic illnesses, as well as their family caregivers, during the patient’s transition from hospital to home [5, 6]. Several studies have shown that high-quality transitional care can ensure patient safety and significantly improve clinical outcomes, such as reduced patient hospitalization and readmission times, emergency visits, lower healthcare costs, and prolonged survival time [7–9].

However, high-quality transitional care is not automatic, it necessitates continuous monitoring and assessment to drive improvement [10]. Numerous studies have demonstrated that patients are particularly vulnerable during transitions from different settings or between levels of care [5, 11, 12]. The transition from hospital to home poses a significant challenge for elderly patients with chronic diseases [4]. Currently, most domestic research focuses on developing various intervention models for transitional care specific to different chronic diseases and validating their effectiveness [13–15]. Few studies have specifically addressed the quality of transitional care as patients move from hospital to home. Therefore, this study aims to investigate the quality of transitional care in elderly patients with chronic diseases and analyze its influencing factors. We hope that the findings of this research can offer practical guidance for further enhancing the quality of transitional care, improving the medical and healthcare service system, and promoting healthy aging.

## Method

### Design and setting

We used a cross-sectional study design and the Chinese version of PACT-M which can assess the quality of transitional care from the patient’s perspective to evaluate the quality of transitional care [16]. We investigated three affiliated hospitals of Zhengzhou University in Henan Province and two tertiary hospitals in Shanxi Province. The five hospitals we surveyed were all tertiary general hospitals with a comprehensive set of departments, including respiratory, cardiovascular, gastrointestinal, endocrine, neurological, and oncology departments. There were more elderly patients with chronic diseases, and the sample size was sufficient. Some studies have shown that transitional care may be related to patient and family factors, hospital factors, and social factors [17]. Therefore, we selected patients’ general information, the Short Form-8 Health Survey, and the Social Support Rating Scale to analyze the factors affecting the quality of transitional care.

### Sample

The Chinese version of PACT-M includes two parts, PACT-M_1_ and PACT-M_2_. The PACT-M_1_ captures the immediate post-discharge period, and the PACT-M_2_ assesses the longer-term experience of managing health and care at home. We survey in two stages: the first stage used PACT-M_1_ to conduct a face-to-face survey when patients were about to be discharged from the hospital, and the second stage used PACT-M_2_ to conduct a telephone interview with patients who had participated in the first stage of the survey one month after discharge. The sample size for influencing factor analysis in cross-sectional surveys is at least 5 to 10 times the number of variables [18]. There were 12 independent variables in this study, including 10 general information variables, 1 social support variable, and 1 SF-8 variable. We calculated the sample size with 10 times the number of variables and taking into account 20% invalid questionnaires, the study required at least 144 samples. We conducted the survey from August 1st, 2022, to May 30th, 2023, distributed 203 questionnaires, and returned 182 valid questionnaires with a response rate of 89.65%.

The inclusion criteria for patients were as follows: [1] the patients were 60 years of age and older, [2] the patients had one or more chronic diseases and were discharged to home rather than to a long-term care facility, and [3] patients had no significant cognitive or language impairment and were willing to participate in the survey.

### Instruments

#### General information questionnaire

The general information questionnaire included patients’ demographic-social information and disease-related information. The demographic-social information included the patients’ gender, age, marital status, education level, pre-retirement occupation, etc. The disease-related information included the type and number of chronic diseases suffered, medical insurance, caregiver status, and so on.

#### The Chinese version of PACT-M

Our research team developed the Chinese version of the PACT-M. The scale has a total of 17 items, each item was rated a score of 1–5 from “Strongly Disagree” to “Strongly Agree”. The PACT-M_1_ and PACT-M_2_ scores range from 0 to 45 and 0 to 40, respectively. The higher total scores for both parts indicate better quality of transitional care. The scale has good reliability and validity and can be used to assess the quality of transitional care for elderly patients with chronic diseases [6, 16]. The Cronbach’s alpha of the PACT‑M_1_ and PACT‑M_2_ in this study were 0.90 and 0.92.

#### The short Form-8 Health Survey (SF-8)

The SF-8 is a versatile and widely applicable short-form health-related quality-of-life instrument that was developed by the RAND Corporation and the Medical Outcomes Study in the 1980s [19]. It encompasses eight domains: general health, physical functioning, role physical, body pain, vitality, social functioning, mental health, and role emotion. It has proven effective in monitoring population health and conducting large-scale outcome studies. The SF-8 has been translated into over 170 languages and extensively tested across many countries. The Chinese version of SF-8 has also demonstrated feasibility, reliability, and effectiveness. The formula for scoring each item on the SF-8 is Standardized Score= (Actual Score-Lowest Score) / (Highest Score-Lowest Score) × 100, with a higher score indicating better health [20]. The Cronbach’s alpha of the Chinese version of SF-8 in this study was 0.85.

#### The Social Support rating scale

The Social Support Rating Scale was developed by Xiao Shuiyuan in 1986 [21], which included objective support (3 items), subjective support (4 items), and utilization of social support (3 items). The score of the objective support dimension is obtained by summing the scores of the 2nd, 6th, and 7th items. The score of the subjective support dimension is obtained by summing the scores of the 1st, 3rd, 4th, and 5th items. The support utilization dimension is obtained by summing the scores of the 8th, 9th, and 10th items. Of these, items 1–4, and 5–8, choose only one for each item and score 1, 2, 3, and 4 for choosing 1, 2, 3, and 4, respectively. Item 5 includes items A, B, C, D, and E, with each item scoring 1–4 from “none” to “full support” respectively. The score of item 5 was the total of items A to E. For items 6 and 7, if the answer was “no source”, the score was 0; if the answer was “the following sources”, the score was the number of sources. The scale has good reliability and validity [22, 23], the Cronbach’s alpha of this scale in this study was 0.74.

#### Data collection

We carried out the survey by a team of 5 graduate students undergoing unified training. After obtaining patients’ consent and signing the informed consent form, the investigators used uniform language to explain the objectives, content, methodology, and precautions for completing the questionnaire. First, we conducted a face-to-face survey using PACT-M_1_ when patients were nearing discharge from the hospital. Subsequently, we conducted a telephone interview using the PACT-M_2_, SF-10, and the Social Support Rating Scale 1 month after patients’ discharge with those who had participated in the first phase of the survey.

The PACT-M_1_ survey was conducted through face-to-face interviews to ensure data completeness. Following the completion of the questionnaire by the patient, the investigator will check it. In case of any omission, the investigator will communicate with the patient to ascertain the reason, if the omission is due to visibility issues, the investigator will kindly request relevant information from the patient. If the patient didn’t know how to answer, the investigator would provide a detailed instruction and assist in completing the questionnaire. For implementation of the PACT-M_2_ survey, we completed via telephone follow-up, and all patients completed the questionnaire except those who did not answer or were not willing to accept the follow-up.

### Statistical analysis

The questionnaires were collected and numbered uniformly. We used EpiData 3.1 software for systematical logical error checking and SPSS 21.0 for statistical analysis. First, we used kurtosis and skewness to test if the data conforms to a normal distribution. After establishing the data were normally distributed, we used frequency ± standard deviation to evaluate the quality of transitional care, t-test or one-way analysis of variance (ANOVA) to analyze the differences in the quality of transitional care reported by patients with different demographic characteristics, and multivariate regression to analyze the factors affecting the quality of transitional care of elderly patients with chronic diseases.

### Ethical statement

The Zhengzhou University ethics committee in China approved all study procedures (approval number: ZZUIRB2021-78) and all patients provided written consent.

## Results

### Participant characteristics

In this study, we distributed 203 questionnaires, with 182 patients completed the PACT-M (response rate 89.65%). The age range of the patients was between 60 and 91 years. The majority of participants were male (61.5%). The educational level was predominantly low, with 83.0% of participants completed high school or Junior college and below. Most participants (89.0%) resided with their spouses or other family members. Additionally, most patients presented with two or more diseases. Further demographic characteristics are presented in Table 1.

**Table 1:** Participants’ characteristics (*n* = 182)

Variables		*N* = 182	Percent (%)
**Gender**	Female	70	38.5
	Male	112	61.5
**Age**	60~	102	56.0
	70~	63	34.6
	80~	17	9.3
**Marital status**	Unmarried	0	0
	Married	145	79.7
	Divorced	2	1.1
	Widowed	33	18.1
	Remarriage	2	1.1
**Ethnic group**	Han nationality	178	97.8
	Other	4	2.2
**Religion**	With	12	6.6
	Without	170	93.4
**Education level**	Junior high school and below	107	58.8
	High school and Junior college	44	24.2
	Undergraduate and above	31	17.0
**Preretirement occupation**	Worker	33	18.1
	Farmer	79	43.4
	Enterprise (business) unit	54	29.7
	Individual household	12	6.6
	Medical and nursing personnel	2	1.1
	No fixed work	2	1.1
	Town	14	7.7
	Rural	79	4.4
**Residence**	City	89	48.9
**Household income per month (Yuan)**	<1000	57	31.3
	1000~	46	25.3
	3000~	47	25.8
	5000~	32	17.6
**Living situation**	Living alone	20	11.0
	Living with spouse	86	47.3
	Living with children	42	23.1
	Living with spouse and children	30	16.5
	Other	4	2.2
**With or without a caregiver**	With	144	79.1
	Without	38	20.9
**If there is a caregiver, the caregiver is**	Spouse	57	31.3
	Children	77	42.3
	Other	48	26.3
**Medical insurance**	Yes	166	91.2
	No	16	8.8
**Number of diseases**	1	46	25.3
	2	84	46.2
	4	38	20.9
	4 or more	14	7.7

### Patients’ experiences in the preparation for transition PACT-M

The overall scores of PACT-M, PACT-M_1_, and PACT-M_2_ were (58.29 ± 14.06), (30.69 ± 7.87) and (27.59 ± 7.14) respectively. The mean scores for the two dimensions within PACT-M_1_ included Perceived health management support at the hospital (3.54 ± 0.88) and Received information and support at the hospital (3.16 ± 0.99). The mean scores for the two dimensions of PACT-M_2_ were as follows: Perceived health management support at home (3.29 ± 0.93) and Home health management (3.72 ± 0.93). The mean scores for the nine items in PACT-M_1_ ranged from 3.09 to 3.90 and the mean scores for the eight items in PACT-M_2_ ranged from 2.78 to 3.82. The detailed results are presented in Table 2.

**Table 2:** Results of scoring patients with PACT-M

PACT-M	Item	Score$(\overline{\varvec{x}}\pm \mathbf{s})$
**PACT-M**_**1**_	**Patients’ experiences in the preparation for transition**	30.69 ± 7.87
	**Perceived health management support at the hospital**	3.54 ± 0.88
	1. I felt I could ask staff questions about what would happen after going home	3.76 ± 1.13
	2. Before leaving the hospital, I was confident I understood how to manage my medication	3.90 ± 0.95
	3. While I was in hospital, there was someone who I could talk to if I was worried	3.82 ± 0.83
	4. Before leaving the hospital, I felt confident about what to do if my health became worse at home	3.26 ± 1.21
	5. I feel that my concerns regarding my health were addressed before I went home	3.10 ± 1.30
	6. I felt prepared to be at home	3.37 ± 1.36
	**Received information and support at the hospital**	3.16 ± 0.99
	1. While I was in the hospital, staff helped me to prepare for things that I might find difficult when I go back home (such as walking, cooking, showering, shopping, or being in pain)	3.29 ± 1.10
	2. Before leaving the hospital, I understood how to get help (or support) from my community services (e.g., doctors, nurses, and home care staff)	3.09 ± 1.16
	3. Before leaving the hospital, I knew what arrangements had been made to support me at home (for example, home care or community care visits)	3.10 ± 1.27
**PACT-M**_**2**_	**Patients’ experiences with managing their health and care at home**	27.59 ± 7.14
	**Perceived health management support at home**	3.29 ± 0.93
	1. I feel I have the support I need from community health services (e.g., doctors, nurses, and home care staff)	2.78 ± 1.26
	2. I feel confident about managing my health at home	3.09 ± 1.24
	3. I feel that there is someone I can talk to about my worries (for example, health care staff or my family)	3.82 ± 0.81
	4. I know what to do and who to contact if my health gets worse	3.70 ± 1.01
	5. I feel I can now manage my care safely at home	3.03 ± 1.23
	**Home health management**	3.72 ± 0.93
	1. I know who to contact if I have any questions about my health and healthcare	3.73 ± 1.16
	2. I know how to manage my medications	3.81 ± 1.03
	3. I have the necessary support to manage everyday activities (e.g., cooking, cleaning, buying food, showering, walking, and dressing)	3.62 ± 1.06
**PACT-M**		58.29 ± 14.06

### Patient’s health status and social support scores

The SF-8 score of elderly patients with chronic diseases was (38.78 ± 18.92), the social support score was (3.41 ± 0.59), and the scores for each item or dimension are presented in Table 3.

**Table 3:** Patients’ health status and social support scores (*N* = 182)

Instruments	Item/Dimension	Score$(\overline{\varvec{x}}\pm \mathbf{s})$
**SF-8**		38.78 ± 18.92
	GH	23.75 ± 24.25
	PF	27.50 ± 18.50
	RP	43.25 ± 27.25
	BP	43.00 ± 22.00
	VT	33.50 ± 22.75
	SF	45.50 ± 28.25
	MH	45.25 ± 25.25
	RE	48.25 ± 28.50
**The Social Support**		3.41 ± 0.59
	Objective support	3.44 ± 0.84
	Subjective support	4.87 ± 0.91
	Utilization of social support	2.22 ± 0.70

### Univariate analysis results of the Chinese version of PACT-M

The kurtosis value of the PACT-M was − 0.043, the kurtosis value was − 0.826, and the data shows an approximate normal distribution. One study [24] showed that If the absolute value of the kurtosis is less than 10 and the absolute value of the skewness is less than 3, it indicates that although the data is not absolutely normal, it is generally acceptable to be a normal distribution. We used the total scores of PACT-M as the dependent variable, and the demographic data as independent variables for one-way ANOVA or independent samples t-test. The results showed statistically significant differences in the PACT-M among patients with different marital status, ethnicity, religion, residence, education level, preretirement occupation, household income per month, and living situation (*P* < 0.05), and the results are detailed in Table 4.

**Table 4:** Univariate analysis of PACT-M in elderly patients with chronic diseases

Variables	The scores of PACT-M	t or F value	P value
**Marital status**		*F* = 2.765	0.043
Unmarried	0.00 ± 0.00		
Married	59.58 ± 13.25		
Divorced	66.00 ± 0.00		
Widowed	52.64 ± 16.68		
Remarriage	49.00 ± 0.00		
**Ethnic group**		*t* = 4.570	0.034
Han nationality	57.96 ± 14.02		
Other	73.00 ± 5.77		
**Religion**		*t* = 6.361	0.004
With	59.08 ± 14.05		
Without	47.00 ± 8.31		
**Education level**		*F* = 5.131	0.007
Junior high school and below	55.16 ± 13.87		
High school and Junior college	64.00 ± 11.32		
Undergraduate and above	57.52 ± 16.25		
**Preretirement occupation**		*F* = 3.258	0.008
Worker	56.39 ± 11.54		
Farmer	55.00 ± 14.42		
Enterprise (business) unit	63.00 ± 13.84		
Individual household	60.83 ± 13.80		
Medical and nursing personnel	78.00 ± 0.00		
No fixed work	57.00 ± 0.00		
**Residence**		*F* = 3.278	0.040
City	60.98 ± 14.02		
Town	55.43 ± 11.86		
Rural	55.76 ± 14.04		
**Household income per month (Yuan)**		*F* = 7.751	< 0.001
<1000	51.87 ± 15.22		
1000~	60.70 ± 9.19		
3000~	59.04 ± 13.68		
5000~	65.13 ± 14.20		
**Living situation**		*F* = 7.334	< 0.001
Living alone	59.80 ± 11.06		
Living with spouse	61.74 ± 11.75		
Living with children	51.38 ± 14.29		
Living with spouse and children	54.40 ± 16.26		
Other	78.00 ± 13.86		

### Multivariable linear regression analysis results of the Chinese version of PACT-M

We used the PACT-M total score of the patients as the dependent variable while incorporating statistically significant variables from the univariate analysis, social support, and SF-8 total scores as independent variables in the multivariate stepwise regression analysis. The assignment of the independent variables and the method for setting the dummy variables are presented in S Table 1 (see Additional file 1), and the other variables were entered as actual values. Simultaneously, Variance Inflation Factor (VIF) and Tolerance were used to assess multicollinearity among the independent variables, which ranged from 0.118 to 0.921, all greater than 0.1; VIF ranged from 1.085 to 8.440, all below 10.00, indicating an absence of multicollinearity. The results showed that pre-retirement occupation, social support, and health status significantly influenced the quality of transitional care among elderly patients with chronic diseases (*P* < 0.05), explaining a total variation of 63.1%. The results of the multiple regression analysis are presented in Table 5.

**Table 5:** Results of multiple regression analysis

Variables	B	SE	β	t	*P* value
**Constant**	8.441	7.316		1.154	0.250
**Preretirement occupation** **(**medical and nursing personnel**)**	20.155	6.883	0.150	2.928	0.004
**Social Support**	0.935	0.164	0.412	5.697	< 0.001
**SF8**	0.920	0.150	0.405	6.148	< 0.001

R^2^ = 0.631, adjusted R^2^ = 0.584; F = 13.581, *P* < 0.01

## Discussion

The primary objective of this study was to use the Chinese version of PACT-M to evaluate the current status of transitional care quality for elderly patients with chronic diseases in China. The result showed that the total scores of PACT-M were (58.29 ± 14.06), and PACT-M_1_ and PACT-M_2_ were (30.69 ± 7.87) and (27.59 ± 7.14) respectively, and the scores for each item in both dimensions ranged from 2.78 to 3.90. Compared with the PACT-M_1_ and PACT-M_2_ total score ranges of 9 ~ 45 and 8 ~ 40 and each entry scores of 1–5, the PACT-M_1_ and PACT-M_2_ scores of the patients in this study were slightly above the median scores of 27 and 24. The lowest scoring entry was entry 1 in the PACT-M_2_: “I think I have the support I need from community health services (e.g., doctors, nurses, home care staff),” with a score of less than 3, which suggests that the patient received limited transitional care after discharge. The State has introduced a series of policies to encourage healthcare organizations to carry out service coordination, guide and assist patient referrals, and promote the continuity of healthcare services [25, 26]. However, the implementation of an effective hospital-community-family triad mechanism is yet to be achieved [27]. In the process of hospital-community-family triad linkage, community health services are a key link in the effective connection between homes and hospitals. Continuously upgrading the ability of community hospitals to prevent and treat diseases and manage health is not only an inevitable requirement to meet the health needs of the elderly population but also the key to promoting the faster formation of a hierarchical treatment pattern and improving the quality of transitional care [28]. However, China’s investment in medical and health institutions primarily focuses on large hospitals, with relatively inadequate funding allocated to primary medical and health institutions. The construction of some urban community health service centers is not yet sound, with insufficient hardware and facilities, a serious brain drain, and a general lack of capacity in primary health care services [29], all of which result in patients not being able to obtain effective transitional care services after discharge from hospitals, seriously affecting the quality of transitional care. Although all other entries are above 3, there is still much room for improvement. Therefore, it is recommended that hospitals above the second level establish a support mechanism with the community to provide technical support and theoretical guidance through peer-to-peer support, remote training, on-the-job training, etc. By gradually improving the capacity of the community to provide healthcare services for elderly patients, promoting effective linkages among hospitals, communities, and families, and improving the quality of transitional care.

Another aim of this paper is to explore the factors that affect the quality of transitional care. The results of the multiple regression analysis showed that the factors affecting the quality of transitional care include pre-retirement occupation, social support, and the health status of patients. Patients with pre-retirement occupations as healthcare workers experience a higher quality of transitional care than patients with other occupations, this is similar to the findings of Marianne Storm et al [30], who identified patient characteristics and information exchange as the key challenges affecting the quality of transitional care. Most patients in the study had two or more diseases, they still had a lot of health issues to deal with and faced many challenges after discharge from the hospital. Patients and their caregivers need to know how to access professional knowledge, skills, and resources (social support to help individuals manage their illnesses, etc.) to provide care at home and to respond appropriately to the challenges of daily living and unfamiliarity [31]. Those whose pre-retirement occupation was healthcare worker have more medical knowledge, which facilitates disease management after discharge from the hospital, and they also have more accessible healthcare resources and timely access to information related to health management compared to other occupational groups. This suggests that we should focus on elderly patients who have a lack of disease-related knowledge and limited access to resources, which can be achieved by explaining disease management-related knowledge to patients and their families at the time of discharge, as well as educating them on the ways to access healthcare resources, and increasing the number and frequency of return visits so that patients whose occupations were non-healthcare workers before retirement can also receive timely and appropriate transitional care to promote their health.

In addition, the study revealed that the patient’s social support and health status had significant effects on the quality of transitional care. Elderly patients with chronic diseases have complex health issues that may negatively impact their daily lives. They often have a high need for social support, in addition to needing transitional care from healthcare providers, they need more support from family caregivers to help them manage their daily lives [32]. Several studies have demonstrated that social support plays a crucial salutogenic resource for individuals’ mental health [33] and high levels of social support can enhance patients’ health-related health status [34] and protect people from disease and early death [35, 36]. Conversely, If individuals don’t receive appropriate support, can negatively affect an individual’s financial, emotional, and psychological well-being and health-related quality of life [37–39]. In this study, patients with high social support also had a higher perceived quality of transitional care, which may be related to the fact that patients get along well with their family and friends, and are better cared for emotionally and in their lives. We suggest that healthcare professionals should focus on patients with weak social support, such as unmarried, divorced, and widowed patients, and provide them with more social support, such as providing family bed services, increasing the number and frequency of follow-up visits to understand the patient’s condition, and solving their post-discharge health problems, to improve their health status.

### Strengths and limitations

A major strength of this study is that it focuses on the transition from hospital to home: a weak link in China’s healthcare services. We learned about the current status of the quality of transitional care from the patient’s perspective. Furthermore, we have identified significant opportunities for enhancing the quality of transitional care, indicating that the development of community health services can effectively augment post-discharge capacities and ultimately improve patient health outcomes. Another strength is that we have analyzed the factors that influence the quality of transitional care, which can provide a basis for precise interventions in transitional care.

There are several limitations to our study. First, the study was a cross-sectional survey and the participants were from 5 tertiary A-level hospitals in Henan and Shanxi provinces, China. Therefore, the results only show which factors affect the quality of transitional care, but cannot explore the causal relationship between study variables. In addition, the hospitals were all at the top of the list for their level of care. They represent a better level of transitional care and are not representative of the level of transitional care in secondary hospitals or the community. There is also volunteer bias and nonresponse bias in the selection of participants, as well as an increased chance of recall bias in studies using self-reported data. Second, there is no literature on the factors influencing the quality of transitional care for elderly patients with chronic illnesses, we only included patients’ demographic and sociological information, health status, and social support as influencing factors, and whether other factors affect the quality of transitional care needs to be further study. Finally, the study sample size was small and the inclusion criteria excluded elderly patients with cognitive and language impairments or those discharged to other long-term care facilities. Therefore, the representativeness and applicability of the research results are limited.

## Conclusion

The results of this study showed that there is still much room for improvement in the quality of transitional care for elderly chronic disease patients. It is imperative to enhance the hospital-community-family tripartite linkage service, increase the allocation of community resources and training of healthcare personnel, and give full play to the vital role of the community in the three-tier linkage, to provide high-quality transitional care for elderly patients with chronic diseases. Factors affecting the quality of transitional care include pre-retirement occupation, social support, and health status. Elderly patients with chronic diseases have complex conditions and still have a high demand for health services after discharge from the hospital. Healthcare professionals should pay attention to the mastery of knowledge and skills related to disease management of patients and provide them with individualized interventions based on the specific requirements of their needs. Elderly patients with poor health and weak social support require particular attention, healthcare professionals should provide regular follow-up visits to understand patients’ transitional care needs and meet their needs for transitional care after hospital discharge and improve their health.

### Electronic supplementary material

Below is the link to the electronic supplementary material.

Supplementary Material 1

## Data Availability

The datasets used and analyzed during the current study are available from the corresponding author upon reasonable request.

## Abbreviations

PACT-M: Partners at Care Transitions Measure
GH: General health
PF: Physical Functioning
RP: Role Physical
BP: Bodily Pain
VT: Vitality
SF: Social Functioning
MH: Mental Health
RE: Role Emotion
