# Supplementary material for: Measuring the quality of transitional care based on elderly patients’ experiences with the partners at care transitions measure: a cross-sectional survey
Source: BMC Nurs. 2024 Mar 14;23:172. doi: 10.1186/s12912-024-01847-7 (PMC10938706; doi:10.1186/s12912-024-01847-7)
Supplement: Supplementary file 1 — Supplementary Material 1 [file 12912_2024_1847_MOESM1_ESM.docx]

**Additional file 1**

S Table 1 The assignment of independent variables

| **Variables** | **Assignment method** |
| --- | --- |
| **Marital status** | Unmarried(X_1_=0, X_2_=0, X_3_=0, X_4_=0)  Married(X_1_=0, X_2_=0, X_3_=0, X_4_=1)  Divorced(X_1_=0, X_2_=0, X_3_=1, X_4_=0)  Widowed(X_1_=0, X_2_=1, X_3_=0, X_4_=0)  Remarriage(X_1_=1, X_2_=0, X_3_=0, X_4_=0) |
| **Ethnic group** | 1=Han nationality; 2=Other |
| **Religion** | 1=With; 2=Without |
| **Education level** | 1=Junior high school and below;  2=High school and Junior college;  3=Undergraduate;  4=Graduate student and above |
| **Preretirement occupation** | Worker(X_1_=0, X_2_=0, X_3_=0, X_4_=0, X_5_=0)  Farmer(X_1_=1, X_2_=0, X_3_=0, X_4_=0, X_5_=0)  Enterprise (business) unit(X_1_=0, X_2_=1, X_3_=0, X_4_=0, X_5_=0)  Individual household(X_1_=0, X_2_=0, X_3_=1, X_4_=0, X_5_=0)  Medical and nursing personnel(X_1_=0, X_2_=0, X_3_=0, X_4_=1, X_5_=0)  No fixed work(X_1_=0, X_2_=0, X_3_=0, X_4_=0, X_5_=1) |
| **Residence** | City(X_1_=0, X_2_=0)  Town(X_1_=1, X_2_=0)  Rural(X_1_=0, X_2_=1) |
| **Household income per month (Yuan)** | 1=＜1000; 2=1000~; 3=3000~; 4=5000~ |
| **Living situation** | Living alone(X_1_=0, X_2_=0, X_3_=0, X_4_=0)  Living with spouse(X_1_=1, X_2_=0, X_3_=0, X_4_=0)  Living with children(X_1_=0, X_2_=1, X_3_=0, X_4_=0)  Living with spouse and children(X_1_=0, X_2_=0, X_3_=1, X_4_=0)  Other(X_1_=0, X_2_=0, X_3_=0, X_4_=1) |
